# A method of correction for heaping error in the variables using validation data

**DOI:** 10.1007/s00362-023-01405-4

**Published:** 2023-02-21

**Authors:** Amar S. Ahmad, Munther Al-Hassan, Hamid Y. Hussain, Nirmin F. Juber, Fred N. Kiwanuka, Mohammed Hag-Ali, Raghib Ali

**Affiliations:** 1 New York University, Abu Dhabi, UAE; 2 Higher Colleges of Technology, Dubai Men’s College, Abu Dhabi, UAE; 3 Data Analysis, Research & Studies Department, Dubai Health Authority, Dubai, UAE; 4 Higher Colleges of Technology, Abu Dhabi, UAE

**Keywords:** Self-reported data, Measurement error, Heaping error, Bias

## Abstract

When self-reported data are used in statistical analysis to estimate the mean and variance, as well as the regression parameters, the estimates tend, in many cases, to be biased. This is because interviewees have a tendency to heap their answers to certain values. The aim of the paper is to examine the bias-inducing effect of the heaping error in self-reported data, and study the effect on the heaping error on the mean and variance of a distribution as well as the regression parameters. As a result a new method is introduced to correct the effects of bias due to the heaping error using validation data. Using publicly available data and simulation studies, it can be shown that the newly developed method is practical and can easily be applied to correct the bias in the estimated mean and variance, as well as in the estimated regression parameters computed from self-reported data. Hence, using the method of correction presented in this paper allows researchers to draw accurate conclusions leading to the right decisions, e.g. regarding health care planning and delivery.

## Introduction

In practice, when interviewees are questioned for data collection they usually do not give true values (Devaux and Sassi 2016). This may be for many reasons; misunderstanding the question, estimating an answer, or feeling uncomfortable giving the most accurate answer (Rosenman et al. 2011). Instead, they may underestimate or exaggerate their responses to certain target values, e.g. nearest integer or to a specific number such as those ending in “zero” or “five” (Pardeshi 2010). So, if one analyses data collected by personal interview then one can note some accumulations on specific values. These given values are also called “target values” (Camarda et al. 2017). An example can be clearly seen in the breastfeeding duration data, where 6, 12, 18 and 24 months are the most frequently reported breastfeeding times (Bracher and Santow 1982; Haaga 1988; Klerman 1993). Another example is data on length of unemployment in months, with 6, 12, 18 and 24 months being the most commonly reported length of unemployment (Neels 2000). In the modern statistical literature, these attractive values are called heaped points and data that lie on these heaped points are called heaped data (Zinn and Würbach 2016). Furthermore, heaping error-in-the-variable can bias the estimates of the mean and variance as well as regression parameters (Wang et al. 2012; Hanisch 2005).

The phenomena of heaping has been intensively investegated in several studies. A novel statistical approach is described that allows us to deal with self-reported heaped income data, where modeling heaping mechanisms and the true underlying model in combination are proposed using the zero-inflated log-normal distribution (Zinn and Würbach 2016). Unemployment duration data was modelled using a maximum likelihood framework based on external validation information, which shows that parameter estimates in discrete-time proportional hazard models of unemployment duration are affected by the heaping mechanism (Kraus and Steiner 1998). A Bayesian approach was implemented by using the Gibbs sampler to reduce biased estimates due to heaping error in measurements from ultrasound images (Wright and Bray 2003). Unemployment spells drawn from the GSOEP West regarding heaping error was examined, and heaping effect on the estimate of a discrete time proportional hazard model was discussed and maximum likelihood approach was used to model the effect of heaping validation (Kraus and Steiner 1998).

Similarly when considering income, the phenomenon of heaping is also apparent and Taiwanese firms have observed that their employees describe their monthly earnings to the nearest 5 within the first two digits of the earnings numbers (Lin et al. 2011). The possible effects of heaping on the parameter estimates of an exponential model a Weibull and a log-logistic model was examined by Monte Carlo simulations and shown that heaping phenomena is common and may impact the relationship between reported duration of unemployment and the actual duration (Torelli and Trivellato 1993).

In the same way, the bias for general cluster patterns was examined and recommendations for bias correction were obtained. The use of Monte-Carlo simulations shows that the amount of heaping that characterizes the GSOEP West does not result in significantly biased parameter estimates of a Weibull model. However, this clearly leads to false seasonal effects (Wolff and Augustin 2003). Self-reported smoking data, specifically number of cigarettes smoked daily, are also subject to a user-dependent heaping bias. Here, multiples of 5 10 or 20 cigarettes smoked daily are reported. Furthermore, it has been shown that the heaping error in this type of data can bias the estimation of parameters of interest such as mean cigarette consumption (Wang and Heitjan 2008).

It has been suggested that heaping adds significantly to the distortion of self-reported number of cigarettes smoked daily (Wang et al. 2012). It has been shown that simple random heaping may lead to a biased estimate of mean daily cigarette consumption of up to 20% depending on both the true mean and the extent of heaping (Wang and Heitjan 2008; Wang et al. 2012). The same heaping errors are present in accurately measured datasets such as birthweight, when births are formally registered. It has been observed that rather than using the birthweight given to at least two decimal places, birthweights are rounded up to the closest integer at the time of birth registration (Barreca et al. 2011).

Multiple imputations are suggested to be used to replace heaped data. This statistical analysis may lead to more accurate estimates for heaping error model misspecification (Heitjan and Rubin 1990). Similalrly, Bayesian hierarchical model was used to control the heaping procedure in longitudinal self-reported counts of sex partners in a study of high-risk behavior in HIV-positive youth (Crawford et al. 2015). The performance of the naive estimators by ignoring the cumulative error for special cases of the Weibull model was examined and a correction approach was introduced when bias due to heaping errors is identified (Augustin and Wolff 2004). An asymmetric rounding, which is a special kind of heaping error in the variables, is investigated. It has neen shown that both symmetric and asymmetric rounding error in the variables has no effect on the expected value, but rather does affect the variance of estimated from rounded data (Schneeweiss et al. 2010).

Section 2 discusses several datasets where both self-reported and measured values are available. Section 3 provides an overview of the self-reported data and the heaping error, which is the difference between the self-reported and measured data. In Sect. 4, we present a method of correction for the bias in the mean and the variance caused by the heaping error in self-reported data. Section 5 is a simulation study and Sect. 6 is the discussion.

## Investigating dataset with self-reported and measured variables

In order to gain practical knowledge about the heaping error, we examine two datasets where both self-reported and measured values are available. The heaping error was calculated as the difference between self-reported and measured values.

### Self-reported and measured sleep duration

Sleep data from 647 subjects (Lauderdale et al. 2008) were collected between 2003 and 2005 using self-reports and measured sleep duration. Objective sleep duration data (in hours) were measured with wrist actigraphy. The mean (standard deviation) of the objective sleep duration and the subjective sleep duration (self-reported) were 6.06 (1.16) and 6.83 (1.11), respectively. This difference of 0.77 between the two means is a simple estimate of bias in the mean. The result of this study shows that the estimated mean of self-reported sleep duration is greater than the estimated mean of measured sleep duration as the mean of the differences (heaping error) is not zero (i.e. bias in the mean).

### Self-reported and measured height, weight and body-mass-index

We investigated a dataset published by Krul et al. (2010) to achieve practical knowledge about the heaping error. The (Krul et al. 2010) dataset includes both self-reported and measured height and weight from 1257 Dutch adults (males and females). The body-mass-index (BMI) was computed as the ratio of measured or reported weight (*kg*) to height ($m^{2}$). Figure 3 shows the distribution of the self-reported and measured height and weight from 1257 Dutch adults (males and females) respectively. Figure 3 shows that for the reported body height, the value of 165, 170, 175, 180, 185, 190, and 195 were often given as target values. Whereas, for the reported body weight, the value of 65, 70, 75, 80, 85 and 90 were often given as target values (Fig. 3). These target values (most frequently given values) are seen as attractive heaping values. Furthermore, the heaping error was computed as the difference between the self-reported and measured data. The mean (95% confidence interval) of the heaping error of the height, weight and BMI was estimated with 1.041 (95% CI 0.920, 1.161), $-$ 1.064 (95% CI $-$ 1.243, $-$ 0.886) and $-$ 0.658 (95% CI $-$ 0.724, $-$ 0.592) respectively. The result of this study shows that the estimated mean from self-reported data is a biased estimate of the mean true mean (as compared to the mean estimated from measured data) as the mean of the differences (heaping error) is not zero. Figure 4 presents the density of the heaping error, which is the difference between the self-reported and measured weight (kg), for six targeted values (65, 70, 75, 80, 85 and 90).

**Table 1:** Estimated variance of *X*, $X^{*}$, and $\varDelta$ as well as the estimated covariance between *X*, $X^{*}$ and $\varDelta$ correspondingly

Estimate	Height	Height
${\mathbb {V}}X$	109.4	276.9
${\mathbb {V}}X^{*}$	112.9	260.6
${\mathbb {V}}\varDelta$	4.741	10.41
${\mathbb {C}}ov(X,\varDelta )$	$-$ 0.66	$-$ 13.35
${\mathbb {C}}ov(X^{*},\varDelta )$	4.081	$-$ 2.943
${\mathbb {C}}ov(X,X^{*})$	108.8	263.9

Furthermore, the estimated variance of self-reported error is 4.7 and 10.4 for the height and weight variables. Whereas, the measured (self-reported) height and weight was 109.4 (112.9) and 276.9 (260.6) respectively. We can see from this dataset that the estimated variance of the height and weight from self-reported data is a biased estimate of the true variance. Moreover, the variance of the measured height is a grater than the variance of the self-reported height. However, the variance of the measured weight is less than the variance of the self-reported weight. Table 1 shows the estimated variance of measured, self-reported and the differences (self-reported minus measured) height and weight; as well as the estimated covariance between measured, self-reported and the differences for height and weight respectively.

Data from 168 students were included in a study to validate on-line self-reported heights and weights against objectively measurements (Nikolaou et al. 2017). The estimated mean (SD) self-reported and measured weight was 66.9 (17.7) kg and 67.5 (16.7) kg respectively. The estimated mean (SD) of the differences between self-reported and measured weight was $-$ 0.6 (0.54) kg. Furthermore, after rounding to two decimal numbers, the estimated mean (SD) self-reported and measured height were the same 1.71 (0.09) m and 1.71 (0.07) m, respectively. The computed BMI calculated from self-reported height and weight was significantly lower than measured, by 0.2 (0.2) kg/m$^2$.

The accuracy of self-reported height and body mass measuremnts were compared to measured values within the US law enforcement population, and the impact these estimations have on the accuracy of BMI classifications (Dawes et al. 2019). Data from 33 male law enforcement officers (age: $40.48 \pm 6.66$ years) were included in the study. The estimated mean (standard deviation) of measured height and body mass was 180.42 (6.87) cm 100.82 (19.86) kg respectively. However, the estimated mean (standard deviation) of reported height and body mass was 180.49 (6.62) cm and 100.59 (19.54) kg correspondingly. Self-report bias in the mean, which is the estimated mean (standard deviation) of differences betweeen self-reported and measured height and body mass was 0.08 (0.25) cm and $-$ 0.23 (0.32) kg respectively.

**Table 2:** Mean differences between self-reported and measured heights (cm) and weight (kg) by survey year for females (F) and males (M): National Health and Nutrition Examination Survey, 1999–2016

Year	Weight-F	Weight-M	Height-F	Height-M
1999–2000	$-$ 1.55	0.15	0.63	1.07
2001–2002	$-$ 1.47	0.20	0.59	1.06
2003–2004	$-$ 1.17	0.18	0.75	1.28
2005–2006	$-$ 1.33	$-$ 0.04	0.76	1.35
2007–2008	$-$ 1.37	0.01	0.85	1.41
2009–2010	$-$ 1.27	$-$ 0.25	1.00	1.34
2011–2012	$-$ 1.35	$-$ 0.02	0.99	1.31
2013–2014	$-$ 1.38	$-$ 0.30	0.97	1.47
2015–2016	$-$ 1.46	$-$ 0.52	1.20	1.81

Flegal et al. (2019) compared national estimates of self-reported and measured height and weight among adults from three US surveys; namely, the National Health and Nutrition Examination Survey (NHANES) for the years 1999 to 2016. Table 2 shows mean differences between self-reported and measured heights (cm) and weight (kg) by survey year for females and males (NHANES), 1999–2016. From Table 2 one can see that mean of self-reported data is not equal to the mean of measured data and the calculated mean differences represent the estimated bias in the mean.

## Overview

Let *X* be a continuous random variable with density $f(x)=f(x,\vartheta )$, where $\vartheta$ is an unknown parameter with a parameter space $\varOmega$. Moreover, let $X^{*}$ be the self-reported variable for the unmeasured variable *X*. The self-reported variable $X^{*}$ can be written as1where $\varDelta$ is the measurement error, which is the difference between the self-reported variable and unmeasured variable ($\varDelta = X^{*} - X$). Equation (1) looks similar to the classical measurement error model. However, the measurement error $\varDelta$ is not independent of *X*. Instead of that, $\varDelta$ is a function of *X* as $X^{*}$ is a function of *X* (Augustin and Wolff 2004; Schneeweiss et al. 2010).

Based on our practical knowledge and several simulations as well as the data we discuss in Sect. 2, we define a new probabilistic heaping model that shows how data can be self-reported in practice. Here, we assume that the unmeasured variable *X* in (1) is heaped with multiple target values or rounded according to

2 $$\begin{aligned} X^{*}= \left\{ \begin{array}{ll} a_{j^{*}}\quad \hbox {if} \quad B<\displaystyle {\max _{j}}\,\, HP_{j}\\ round(X)\quad \hbox {if} \quad B\ge \displaystyle {\max _{j}}\,\, HP_{j} \end{array} \right. \end{aligned}$$

with

$$\begin{aligned} HP_{j}(X)=\exp \left( \frac{-\sqrt{|X-a_{[j]}|}}{I}\right) \end{aligned}$$

where $j^{*}=\arg \displaystyle {\max _{j}}\,\, HP_{j}$, *I* is a predetermined intensity, *round*() is the simple rounding function and *B* is a uniformly distributed random variable on the interval [0; 1] ($B\sim U[0;1]$), with

$$\begin{aligned} a_{1}<a_{2}<\cdots<a_{j}<\cdots <a_{k}. \end{aligned}$$

**Fig. 1:**
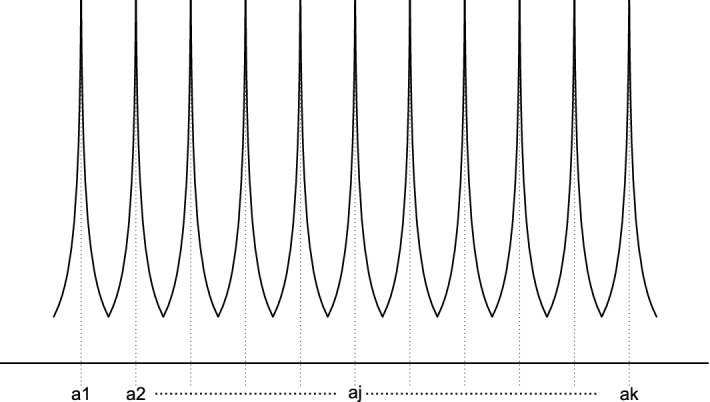
Heaping profiles with multiple target values

Figure 1 shows heaping profiles with multiple target values, which is similar to Fig. 4 and Figure 1 in Torelli and Trivellato (1993). Note that $X^{*}=X$ in Eq. (2), if $B\ge \displaystyle {\max _{j}}\,\, HP_{j}$ and *X* is a discrete random variable. Equation (2) allows for overlapping, i.e. values can be heaped to different heaping points. For example, a weight value of 68.93, 69.12, 70.88, 72.36, 74.27, and 75.29 can be heaped to 70.

Through repeated multiple different simulations and investigating several datasets (see Sect. 2), we have found out that the estimated mean and variance of the self-reported variable $X^{*}$ is, in many cases,^1^ a biased estimator of the mean and variance of the *X* variable. Therefore, we have under Eq. (2)

3 $$\begin{aligned} {\mathbb {E}}X^{*} = {\mathbb {E}}(X+\varDelta ) = {\mathbb {E}}X + \underbrace{{\mathbb {E}}(\varDelta )}_{\ne 0} \ne {\mathbb {E}}X \end{aligned}$$

(see Tables 2 and 4). Furthermore,

4 $$\begin{aligned} {\mathbb {V}}X^{*}= & {} {\mathbb {V}}(X+\varDelta )\nonumber \\= & {} {\mathbb {V}}X + {\mathbb {V}}\varDelta + 2\underbrace{{\mathbb {C}}ov(X,\varDelta )}_{\ne 0} \end{aligned}$$

The variance of the self-reported data is not equal to the variance of the measured data. However, the variance of the self-reported data can be greater than or less than the variance of the measured. This depends on the data we are investigating and how the data were self-reported. For example, the variance of the self-reported data can be less or greater than the variance of the measured data, if ${\mathbb {V}}\varDelta + 2{\mathbb {C}}ov(X,\varDelta ) > {\mathbb {V}}X$ or ${\mathbb {V}}\varDelta + 2{\mathbb {C}}ov(X,\varDelta ) < {\mathbb {V}}X$ correspondingly (see Tables 1 and 4).

## Method of correction

Assuming that we interview *N* participants to collect a certain amount of information (for example their weight). The answers $X^{*}$ of these *N* interviewees will not be accurate and usually heaped or rounded to an attractive number or to the nearest integer. Therefore, we randomly select a subsample *n* from these *N* participants to exactly measure the required information.

We assume an approximately linear relationship between these two variables according to the following

5 $$\begin{aligned} X = X^{*}\beta +\epsilon \end{aligned}$$

with

$$\begin{aligned} \beta = \left( \begin{array}{cccc} \displaystyle {\beta _{0}}\\ \displaystyle {\beta _{1}} \end{array} \right) ,\quad X = \left( \begin{array}{cccc} \displaystyle {1} &{}\displaystyle {x_{1}}\\ \displaystyle {1}&{}\displaystyle {x_{2}}\\ \vdots &{} \vdots \\ \displaystyle {1}&{}\displaystyle {x_{n}} \end{array} \right) ,\hbox { and}\quad X^{*} = \left( \begin{array}{cccc} \displaystyle {1} &{}\displaystyle {x_{1}^{*}}\\ \displaystyle {1}&{}\displaystyle {x_{2}^{*}}\\ \vdots &{} \vdots \\ \displaystyle {1}&{}\displaystyle {x_{n}^{*}} \end{array} \right) . \end{aligned}$$

A linear regression assume that the conditional expectation value of the residual $\epsilon$ given an exogenous observed variable should equal zero. Even if this is not satisfies, the linear model in (5) is still a good approximation between *X* and $X^{*}$. In general a linear model assumes that

6 $$\begin{aligned} {\mathbb {E}}(\epsilon |X^{*})= 0, \end{aligned}$$

However, in the case of heaped data the true variable *X* and heaping error $\varDelta$ are assumed to be dependent. Thus, on the first consideration, one has to ask whether the assumption in (6) may break down. But through repeated multiple different simulations we have found out that ${\mathbb {E}}(X|X^{*})\approx X^{*}\beta$. Therefore

$$\begin{aligned} {\mathbb {E}}(\epsilon |X^{*})\approx 0, \end{aligned}$$

Hence, we estimate the unknown parameters $\beta$ by the method of least squares, which consists of finding the values of

$$\begin{aligned} {\widehat{\beta }} = \left( X^{*\top }X^{*}\right) ^{-1}X^{*\top }X, \end{aligned}$$

that minimize the sum of squares of the residuals

$$\begin{aligned} {\widehat{\epsilon }}= X-X^{*}{\widehat{\beta }}. \end{aligned}$$

We estimate the unmeasured values of the *X* variable for the remaining $N-n$ observation according to

$$\begin{aligned} {\widehat{X}} = {\widehat{W}} + u \end{aligned}$$

where ${\widehat{W}}=X^{*}{\widehat{\beta }}$ and *u* is simulated normal distributed random variable with mean zero and variance $\sigma _{u}^{2} = \sigma _{{\widehat{\epsilon }}}^{2}$.

Here we assume that $\sigma _{u}^{2} = \sigma _{{\widehat{\epsilon }}}^{2}$, because we have selected our subsample randomly, therefore the estimated variance of the residuals $\sigma _{{\widehat{\epsilon }}}^{2}$ is representative for variance of the residuals of the whole sample.

Then, we have a new variable ${\widetilde{X}}$ and ${\widetilde{W}}$ such as

7 $$\begin{aligned} {\widetilde{W}}_{j}=\left\{ \begin{array}{ll} X_{{\tiny {\hbox {obs}}}j}&{}\quad \hbox {if} \quad j\in n\\ {\widehat{W}}_{j}&{}\quad \hbox {otherwise}. \end{array} \right. \end{aligned}$$

and

8 $$\begin{aligned} {\widetilde{X}}_{j}=\left\{ \begin{array}{ll} X_{{\tiny {\hbox {obs}}}j}&{}\quad \hbox {if} \quad j\in n\\ {\widehat{X}}&{}\quad \hbox {otherwise}, \end{array} \right. \end{aligned}$$

### Comparison of the mean and the variance

We use validation data to estimate ${\tilde{X}}$, we would like to prove that the newly estimated variable ${\tilde{X}}$ has an equal mean and variance to the mean and variance of unmeasured variable *X*.

#### Proof

From (8) we have

$$\begin{aligned} {\mathbb {E}}({\widetilde{X}}_{j})= & {} \left\{ \begin{array}{ll} {\mathbb {E}}(X_{{\tiny {\hbox {obs}}}j})={\mathbb {E}}X&{}\quad \hbox {if} \quad j\in n\\ {\mathbb {E}}({\widehat{X}}_{j})&{}\quad \hbox {otherwise} \end{array} \right. \end{aligned}$$

with

9 $$\begin{aligned} {\mathbb {E}}({\widehat{X}}_{j})= & {} {\mathbb {E}}({\widehat{W}}_{j} + u)\nonumber \\= & {} {\mathbb {E}}(X^{*}{\widehat{\beta }} + u)\nonumber \\= & {} {\mathbb {E}}(X^{*}{\widehat{\beta }}) + \underbrace{{\mathbb {E}}(u)}_{=0}\nonumber \\= & {} {\mathbb {E}}(X^{*}{\widehat{\beta }})\nonumber \\= & {} {\mathbb {E}}(X^{*}){\mathbb {E}}({\widehat{\beta }})\nonumber \\= & {} {\mathbb {E}}(X^{*})\beta \nonumber \\= & {} {\mathbb {E}} X \end{aligned}$$

$$\therefore $$ $${\mathbb {E}}({\widetilde{X}})={\mathbb {E}}(X)$$. $$\square $$

#### Proof

Furthermore

$$\begin{aligned} {\mathbb {V}}({\tilde{X}}_{j})= & {} \left\{ \begin{array}{ll} {\mathbb {V}}(X_{{\tiny {\hbox {obs}}}j})={\mathbb {V}}X&{}\quad \hbox {if} \quad j\in n\\ {\mathbb {V}}({\widehat{X}}_{j})&{}\quad \hbox {otherwise} \end{array} \right. \end{aligned}$$

with

$$\begin{aligned} {\mathbb {V}}({\widehat{X}}_{j})= & {} {\mathbb {V}}(W_{j}^{*}{\widehat{\beta }} + u)\\= & {} {\mathbb {V}}(X_{j}^{*}{\widehat{\beta }} + u)\\= & {} {\mathbb {V}}(X_{j}^{*}{\widehat{\beta }}) + {\mathbb {V}}(u) \end{aligned}$$

this because *u* is independent of ${\widehat{\beta }}$ and $X^{*}$.

Let

$$\begin{aligned}{} & {} {\underline{X}} = \left( \begin{array}{cccc} \displaystyle {1}\\ \displaystyle {X_{j}^{*}} \end{array} \right) ,\,\, \mu = \left( \begin{array}{cccc} \displaystyle {1}\\ \displaystyle {\mu } \end{array} \right) ,\,\, \varSigma _{X} \left( \begin{array}{cccc} \displaystyle {0} &{}\displaystyle {0}\\ \displaystyle {0}&{}\displaystyle {\sigma _{X}^{2}} \end{array} \right) , \,\, \beta = \left( \begin{array}{cccc} \displaystyle {\beta _{0}}\\ \displaystyle {\beta _{1}} \end{array} \right) ,\,\,\hbox {and} \\ {}{} & {} M = {\mathbb {E}}({\underline{X}}\,\,{\underline{X}}^{\top }), \end{aligned}$$

$$\Longrightarrow $$

$$\begin{aligned} {\mathbb {V}}({\underline{X}}^{\top }{\widehat{\beta }})= & {} {\mathbb {E}}\left[ ({\underline{X}}^{\top }{\widehat{\beta }})^{2}\right] - \left[ {\mathbb {E}}({\underline{X}}^{\top }{\widehat{\beta }})\right] ^{2}\\= & {} {\mathbb {E}}({\widehat{\beta }}^{\top }M{\widehat{\beta }}) - \beta ^{\top }\mu \mu ^{\top }\beta \\= & {} \hbox {tr}\left[ M(\varSigma _{{\widehat{\beta }}} + \beta \beta ^{\top })\right] - \beta ^{\top }\mu \mu ^{\top }\beta \\= & {} \hbox {tr}(M\varSigma _{{\widehat{\beta }}})+ \beta ^{\top }(M-\mu \mu ^{\top })\beta \\= & {} \hbox {tr}(M\varSigma _{{\widehat{\beta }}})+ \beta ^{\top }\varSigma _{X}\beta \\= & {} \hbox {tr}(M\varSigma _{{\widehat{\beta }}})+ \sigma _{X}^{2}\beta _{1}^{2} \end{aligned}$$

$$\hbox {tr}(M\varSigma _{{\widehat{\beta }}})\approx 0$$, because $$\varSigma _{{\widehat{\beta }}}\longrightarrow 0$$ for $$N\longrightarrow \infty $$

$$\Longrightarrow $$

$$\begin{aligned} {\mathbb {V}}(X_{j}^{*}\widehat{\beta _{1}})\approx \beta _{1}^{2}{\mathbb {V}}(X_{j}^{*}) \end{aligned}$$

$$\Longrightarrow $$

10 $$\begin{aligned} {\mathbb {V}}({\widehat{X}}_{j})\approx & {} {\mathbb {V}}(\beta _{1}\cdot X_{j}^{*}) + {\mathbb {V}}(u)\nonumber \\= & {} {\mathbb {V}}(X_{j}^{*}\cdot \beta _{1}) + {\mathbb {V}}(\epsilon )\nonumber \\= & {} {\mathbb {V}}(X) \end{aligned}$$

$$\therefore $$ $${\mathbb {V}}({\tilde{X}})={\mathbb {V}}(X)$$. $$\square $$

Note that the approximation sign ($\approx$) in (10) will be an exact result once the validation data sample size goes to infinity ($n\rightarrow \infty$).

From (9) and (10) we have proved that the mean and the variance of the newly estimated variable ${\tilde{X}}$ are equal to the mean and the variance of the unmeasured variable *X*.

### The influence of heaping on regression estimates

Next, we would like to discuss the influence of self-reported data on the estimates of the regression coefficients. Let *Y* be a measured outcome (dependent variable), and $X^{*}$ and *X* be the self-reported and measured explanatory variable respectively. We consider a simple linear regression model

11 $$\begin{aligned} Y = \alpha _{0} + X\alpha _{1} + \epsilon \end{aligned}$$

where $\alpha _{0}$ and $\alpha _{1}$ are the unknown intercept and slope respectively. The corresponding regression for self-reported and corrected data is $Y = \alpha _{0}^{*} + X^{*}\alpha _{1}^{*} + \epsilon ^{*}$ and $Y = {\tilde{\alpha }}_{0} + {\widetilde{X}}{\tilde{\alpha }}_{1} + {\tilde{\epsilon }}$ respectively.

The true regression parameter $\alpha _{1}$ is estimated by ${\mathbb {C}}ov(X,Y)/{\mathbb {V}}X$, whereas the regression parameter computed from the self-reported data $\alpha _{1}^{*}$ is estimated by ${\mathbb {C}}ov(X^{*},Y)/{\mathbb {V}}X^{*}$, which is differs from $\alpha _{1}$. Furthermore, using the corrected variable ${\widetilde{X}}$ to estimated the unknown parameter $\alpha _{1}$ (${\mathbb {C}}ov({\widetilde{X}},Y)/{\mathbb {V}}{\widetilde{X}}$) will also lead to a biased estimate, although that ${\mathbb {E}}X={\mathbb {E}}{\widetilde{X}}$, and ${\mathbb {V}}X={\mathbb {V}}{\widetilde{X}}$ (when $n\rightarrow \infty$ ). That’s because, under Eq. (2), ${\mathbb {E}}(XY)\ne {\mathbb {E}}({\widetilde{X}},Y)$. Nevertheless, we can estimate an approximately unbiased estimate for $\alpha _{1}$ in Eq. (11) by performing a linear regression mode with ${\widetilde{W}}$ as a predictor of *Y*.^2^

## Simulation study

### Simulation study based on Krul et al. (2010) data

To show the accuracy of the method in Sects. 4, we performed a simulation study with 10,000 replications. First, we used the estimated mean (77.8) and standard deviation (16.6) of the measured weight from Krul et al. data (Krul et al. 2010), to simulate a normally distributed variable *X*, with mean of 77.8 and standard deviation of 16.6 ($X\sim N(77.8, 16.6)$). Then, we heaped-and-rounded the *X* variable into $X^{*}$ as in Eq. (2). For each simulation, we varied the percentage of heaping (from 10%, to 90%, by 10%) and the rest of the sample was rounded classically with zero decimal place (see Eq. (2)). The heaping values (target values) were 65, 70, 75, 80, 85, and 90.

Next, we simulated a simple linear regression model with a normally distributed independent variable *X*, the regression parameters were $\alpha _{0}=-2$ and $\alpha _{1}=2$ and the error term was simulated to be standard normally distributed with mean zero and variance one ($\epsilon \sim N(0,1)$). The dependent variable *Y* was simulated as $Y=\alpha _{1}+\alpha _{1}X+\epsilon$.

Finally, we applied our method of correction in Sect. 4 to correct the mean and variance of the $X^{*}$ variable by estimating the ${\widetilde{W}}$ and ${\widetilde{X}}$ variables as in Eqs. (8) and (7). Next we fitted three simple linear regression models with *Y* as a depended variable and $X^{*}$, ${\widetilde{W}}$ and ${\widetilde{X}}$ as independent variable respectively. The estimates of the regression parameters were computed from 10,000 replications; and the subsample was $n=120$% for each sample size $N=1200$ in each replicate.

**Fig. 2:**
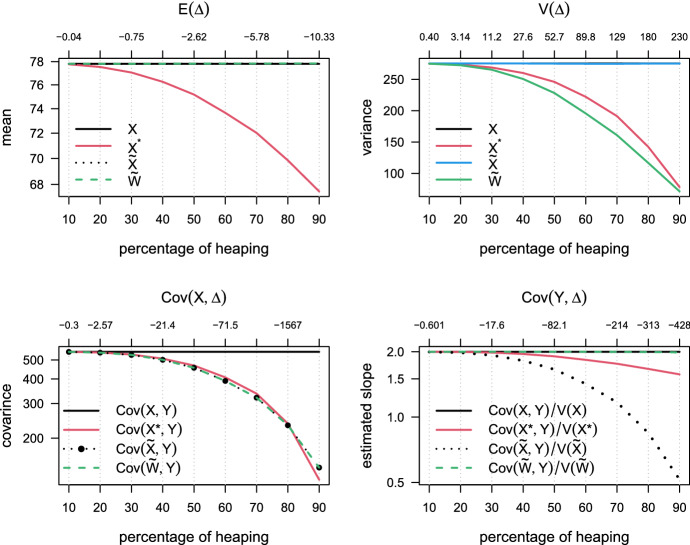
Results of this simulation study in Sect. 5.1. on of 16.6 ($X\sim N(77.8, 16.6)$). Then, we heaped-and-rounded the *X* variable into $X^{*}$ as in Eq. (2). For each simulation, we varied the percentage of heaping (from 10%, to 90%, by 10%) and the rest of the sample was rounded classically with zero decimal place (see Eq. (2)). The heaping values (target values) were 65, 70, 75, 80, 85, and 90

Figure 2 contains four graphs which comprise the results of this simulation study. The top-left graph shows the estimated mean of the measured *X*, heaped-and-rounded $X^{*}$ and corrected variables (${\widetilde{W}}$ and ${\widetilde{X}}$) as well as the mean of the heaping-and-rounded error (${\mathbb {E}}\varDelta$) for different perecetages of heaping. The estimated mean of $X^{*}$ is a biased estimate of the mean of *X* and the difference between ${\mathbb {E}}X^{*}$ and ${\mathbb {E}}X$, which is ${\mathbb {E}}\varDelta$, is exponentially decreasing as the perecetage of heaping increases. However, the estimated mean of the corrected variable ${\widetilde{W}}$ and ${\widetilde{X}}$ is an unbiased estimate of the mean of the true variable *X* (${\mathbb {E}}X={\mathbb {E}}{\widetilde{W}}={\mathbb {E}}{\widetilde{X}}$).

Furthermore, The top-right graph in Fig. 2 demonstrates that the estimated variance of the corrected variable ${\widetilde{X}}$ is an unbiased estimate to the variance of the true variable *X* for all simulated perecetages of heaping (10% to 90%). The estimated variance of the heaping-and-rounded variable $X^{*}$ and corrected ${\mathbb {V}}W$ is a biased estimate of the variance of the measured variable *X* respectively (top-right graph in Fig. 2).

Moreover, The bottom-left graph sindicates that the estimated covariance of the ${\mathbb {C}}ov(X^{*},Y)$, ${\mathbb {C}}ov({\widetilde{W}},Y)$ and ${\mathbb {C}}ov({\widetilde{X}},Y)$ are a biased estimates of the ${\mathbb {C}}ov(X,Y)$; and the degree of bias depends on the degrees of heaping (percentage of heaping). Therefore, the estimated slope by ${\mathbb {C}}ov(Y,X^{*})/{\mathbb {V}}(X^{*})$ and ${\mathbb {C}}ov(Y,{\widetilde{X}})/{\mathbb {V}}({\widetilde{X}})$ is a biased estimate of the true slope ${\mathbb {C}}ov(Y,X)/{\mathbb {V}}(X)$. However, an unbiased estimate of the true slope $\alpha _{1}$ can be estimated by ${\mathbb {C}}ov(Y,{\widetilde{X}})/{\mathbb {V}}({\widetilde{X}})$ (Fig. 2, graph bottom-right).

From Fig. 2, we can see that heaping-and-rounding error leads to biased estimates of the mean and variance of the heaped-and-rounded variable $X^{*}$. This bias increases as the heaping percentage increases. Nevertheless, our method of correction in Sect. 4 can be applied to reach unbiased estimates even.

**Table 3:** Average of the estimated slope from 10,000 simulation from a simple linear model: Regression of *X* on $X^{*}$, *X* on ${\widetilde{X}}$ and *X* on ${\widetilde{W}}$ respectively

(%)	*X* on $X^{*}$	*X* on ${\widetilde{X}}$	*X* on ${\widetilde{W}}$
05	1.000 (0.001)	1.000 (0.002)	1.000 (0.002)
15	0.999 (0.001)	0.996 (0.005)	1.000 (0.004)
25	0.996 (0.003)	0.980 (0.013)	1.000 (0.009)
35	0.985 (0.005)	0.941 (0.024)	1.000 (0.016)
45	0.966 (0.007)	0.876 (0.034)	1.001 (0.024)
55	0.936 (0.011)	0.775 (0.043)	1.002 (0.034)
65	0.902 (0.013)	0.661 (0.048)	1.002 (0.044)
75	0.857 (0.017)	0.509 (0.047)	1.004 (0.056)
85	0.812 (0.022)	0.348 (0.041)	1.004 (0.067)
95	0.766 (0.036)	0.185 (0.033)	0.999 (0.082)

The percentage of heaping was varied, from 5%, to 95%, by 10%

In addition, we simulated $X\sim N(77.8, 16.6)$. Then, we heaped-and-rounded the *X* variable into $X^{*}$ as in Eq. (2). For each simulation, we varied the percentage of heaping (from 5%, to 95%, by 10%) and the rest of the sample was rounded classically with zero decimal place (see Eq. (2)). The heaping values (target values) were 65, 70, 75, 80, 85, and 90. Then we applied our method of correction in Sect. 4 to correct the mean and variance of the $X^{*}$ variable by estimating the ${\widetilde{W}}$ and ${\widetilde{X}}$ variables as in Eqs. (8) and (7). Next we performed three univariate linear regression models with *X* as a dependent variable and $X^{*}$, ${\widetilde{X}}$ and ${\widetilde{W}}$ as an independent variable respectivel. Table 3 shows that the estimated slope of *X* regress on ${\widetilde{W}}$ is approximately equal to one even if 95% of the data were heaped to several heaping points (more than two vaues). However, the estimated slope of *X* regress on $X^{*}$ and ${\widetilde{X}}$ are less than one when 20% of the data are heaped (Table 3).

Through different simulations, we have found out that the linear assumption between self-reported and measured data holds even if 100% of the data were heaped to several heaping points (targeted values). However, the linear assumption between self-reported and measured data breaks down when 100% of the self-reported data are heaped to one or two targeted values. Furthermore, the corrected variable ${\widetilde{W}}$ has the highest correlation with the *Y* variable (between 0.99 to 0.4 for different heaping percentages) compared to the self-reported ($X^{*}$) and corrected ${\widetilde{X}}$ variables.

**Table 4:** Estimated averages of mean and standard deviation (SD) of measured (*X*), self-reported ($X^{*}$), estimated (${\tilde{X}}$) data of the body weight, height and BMI from 10,000 simulations

Mean (SD)			
Sample	Height	Weight	BMI
*X*	174.0 (10.5)	77.8 (16.6)	25.7 (5.0)
$X^{*}$	175.0 (10.6)	76.7 (16.1)	25.0 (4.7)
${\tilde{X}}_{n=126}$	174.0 (10.5)	77.8 (16.6)	25.7 (5.0)
${\tilde{X}}_{n=251}$	174.0 (10.5)	77.8 (16.6)	25.7 (5.0)
${\tilde{X}}_{n=377}$	174.0 (10.5)	77.8 (16.6)	25.7 (5.0)
${\tilde{X}}_{n=503}$	174.0 (10.5)	77.8 (16.6)	25.7 (5.0)
${\tilde{X}}_{n=628}$	174.0 (10.5)	77.8 (16.6)	25.7 (5.0)

Validation datasets with a sample size of n = 126 (10%), 251 (20%), 377 (30%), 503 (40%), and 628 (50%) were used to computed corrected data (${\tilde{X}}$). Note that ${\tilde{X}}_{n=126}$ displays data with sample size N = 1257, using validation data of sample size n = 126 (10% of cohort sample size N = 1257)

**Table 5:** Averages from 10,000 simulations of estimated regression parameters of intercept, age and gender from a bivariate linear regression model with BMI as an outcome

Sample	Intercept	Age	Gender
*X*	19.90	0.155	$-$ 0.422
$X^{*}$	19.70	0.141	$-$ 0.175
${\tilde{X}}_{n=126}$	19.96	0.153	$-$ 0.439
${\tilde{X}}_{n=251}$	19.93	0.154	$-$ 0.425
${\tilde{X}}_{n=377}$	19.90	0.155	$-$ 0.424
${\tilde{X}}_{n=503}$	19.92	0.155	$-$ 0.426
${\tilde{X}}_{n=628}$	19.90	0.155	$-$ 0.425

Note that ${\tilde{X}}_{n=126}$ displays data with sample size N = 1257, using validation data of sample size n = 126 (10% of cohort sample size N = 1257)

### Simulation study using Krul et al. (2010) data

We carried out a simulation study with 10,000 replications to invetegate the accuracy of the method in Sects. 4. We used Krul et al. data (Krul et al. 2010) to compare the mean and the variance of the heaped and measured variable with the mean and the variance of the estimated variable as in (8). Form each simulation (replicate), we randomly sampled (without replacement) a subsample (*n*) from the $N=1257$ measured data (height, weight and BMI). Next we have applied our method of correction in Sect. 4 to estimate the new variable ${\tilde{W}}$ and ${\tilde{X}}$ for the height, weight and BMI respectively. The mean and variance of the estimated height, weight and BMI was estimated from each replicate (simulation) and the average of 10,000 simulations was computed for each variable. We have vary the randomly sampled subsample sizes of 126 (10%), 251 (20%), 377 (30%), 503 (40%) and 628 (50%) respectively. In addition, four bivariate linear regression models were performed with measured, self-reported, and corrected BMI as the outcome. The predictors were age and gender. We estimated the intercept and slopes of each model and for each replicate. Similarly, the sample size (*n*) was varied to 126 (10%), 251 (20%), 377 (30%), 503 (40%), and 628 (50%), respectively.

Table 4 represents the average mean and standard deviation of the height weight and BMI from 10,000 replications. From Table 4 we can see that the mean and standard deviation from the estimated variable ${\tilde{X}}$ are equal to the mean and standard deviation of the measured variable *X* for all validation samples of size (*n*) used in this simulation study. Furthermore, Table 5 shows the average of 10,000 simulations for different subsample sizes of 126 (10%), 251 (20%), 377 (30%), 503 (40%) and 628 (50%) respectively. A good correction for the estimated regression coefficients is obtained, when our method of correction in Sect. 4, is used to estimate the effect of age and gender on BMI as compared with the naive estimators, i.e. when self-reported BMI is used as an outcome variable (Table 5).

## Discussion

In empirical research, variables such as weight (kg) or sleep duration (hours) can be measured directly for each individual (Narciso et al. 2019). However, self-reported measures can be valuable when actual measurements are unavailable or too time consuming or expensive to measure (Short et al. 2009). Self-reported measures can also be very useful in some situations where conducting measurement is not possible, such as during a pandemic (e.g. COVID-19 pandemic). Instead, data can be collected via self-reporting questionnaires (Garcia and Gustavson 1997). Nevertheless, self-reported data are subject to error (Trabulsi and Schoeller 2001), and standard statistical approaches do not consider self-reporting errors in the data. This can, in many cases, leads to invalid inferences.

**Fig. 3:**
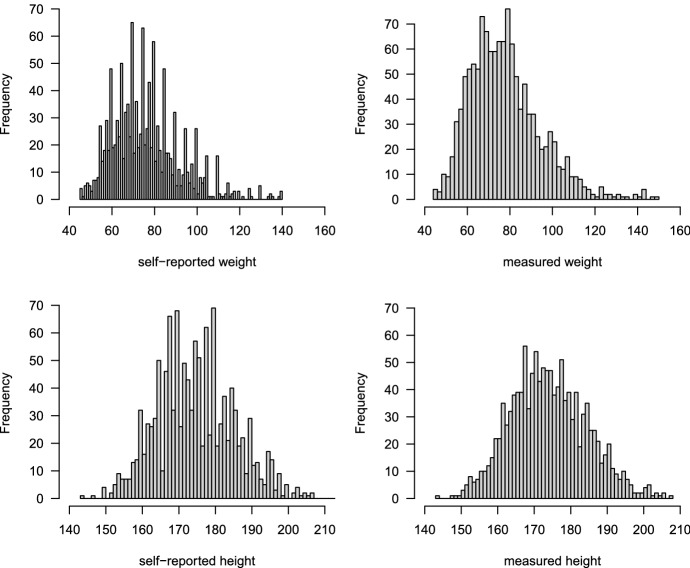
Histogram of the self-reported and measured height and weight

**Fig. 4:**
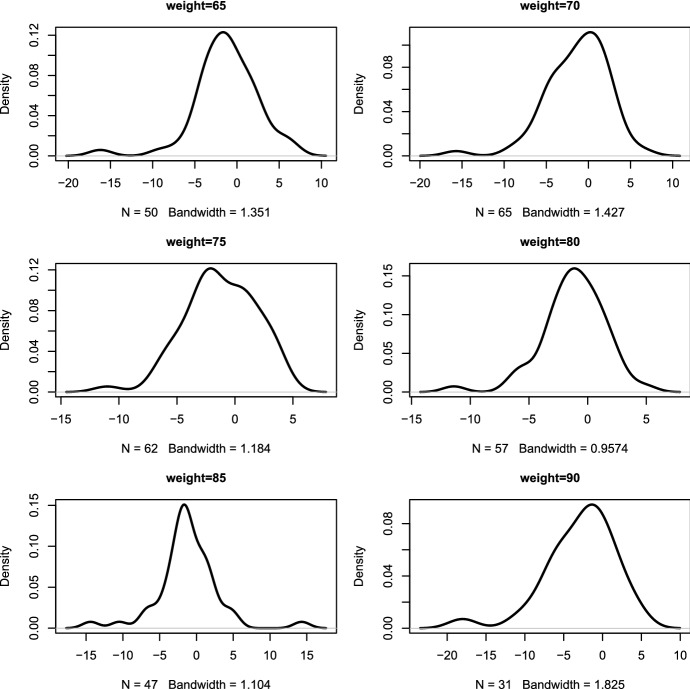
Histogram density of the heaping error for six targeted value of the weight (kg) (65, 70, 75, 80, 85 and 90 heaping values)

This paper investigates to what extent heaping-and-rounding error in self-reported data can affect the estimates of the mean and variance, as well as regression parameters, when self-reported data are used in statistical analysis. Exploring several datasets in Sect. 2, we show that using self-reported data can, in many situation, lead to bias estimates of the mean and variance, as well as regression parameters (Lauderdale et al. 2008; Krul et al. 2010; Dawes et al. 2019; Flegal et al. 2019). This is also because most people are heaping their responses in an asymmetrical manner. For instance, it is well known that many people tend to reduce their real weight (Krul et al. 2010; Short et al. 2009) and income (Maynes 1968) and increase their height (Krul et al. 2010; Short et al. 2009). As a consequence the mean and the variance of the differences between self-reported and measured data are not zero.

As a result, a new method of correction is introduced to correct the heaping error effects using validation data. Validation data are very valuable when self-reported data are collected (Ahmad 2007). In statistics, validation is performed to find out if predicted values from a statistical model are likely to correctly predict responses on future observed values (Frank and Harrell 2015). In our case, the technical term “validation data” denotes an exact measurement of a certain subsample of the data collected at interview time. For example, assuming that we interview *N* persons about a certain amount of information (for example about their height or weight). Their answers are usually heaped, therefore we can randomly select *n* individuals from the study sample size *N* and measure them exactly (without rounding or heaping error). An example of such data can be found in Krul et al. (2010).

Section 5 shows that bias increases as the heaping percentage increases, and that heaping leads to bias in the mean and variance of a heaped variable and this bias depends on the degree of heaping. However, in empirical research, the heaping-and-rounding procedure and the heaping degree are unknown. Nevertheless, our method of correction in Sect. 4 can be applied to reach unbiased estimates even if the the heaping-and-rounding procedure and the heaping degree are unknown. Nevertheless, validation data can be used to make inferences on the mean and variance as well as regression parameters. A randomly selected subsample with a size of 10% of the study sample size *N* is recommended, in order to use our method of correction in Sect. 4. However, a careful consideration should be made to draw a conclusion from the self-reported, validation, and estimated data in (8) and (7). We recommend that a sensitivity analysis should be used where self-reported data of size *N*, validation data of size *n*, and estimated data of size *N* are analyzed.

## Footnotes

1 This is because many people heap their responses in an asymmetrical manner by reducing their real weight and increase their height (Krul et al. 2010).

2 Through several simulations we found that the variable ${\widetilde{W}}$ is perfectly calibrated ($0.999<$ slope $<1.004$) with the measured variable *X*. Also, ${\widetilde{W}}$ has a higher correlation with *Y* compared to $X^{*}$ and ${\widetilde{X}}$.
